# Role of (p)ppGpp in antibiotic resistance, tolerance, persistence and survival in Firmicutes

**DOI:** 10.1093/femsml/uqad009

**Published:** 2023-03-11

**Authors:** Andrea Salzer, Christiane Wolz

**Affiliations:** Interfaculty Institute of Microbiology and Infection Medicine, 72076 Tübingen, Germany; Cluster of Excellence EXC 2124 “Controlling Microbes to Fight Infections”, University of Tübingen, 72076 Tübingen, Germany; Interfaculty Institute of Microbiology and Infection Medicine, 72076 Tübingen, Germany; Cluster of Excellence EXC 2124 “Controlling Microbes to Fight Infections”, University of Tübingen, 72076 Tübingen, Germany

**Keywords:** (p)ppGpp, stringent response, persistence, antibiotic tolerance, antibiotic resistance, stress survival

## Abstract

The stringent response and its signalling nucleotides, pppGpp and ppGpp, have been the subject of intense research since the discovery of (p)ppGpp in 1969. Recent studies have revealed that the downstream events that follow (p)ppGpp accumulation vary among species. Consequently, the stringent response as initially characterized in *Escherichia coli* largely differs from the response in Firmicutes (Bacillota), wherein synthesis and degradation of the messengers (p)ppGpp are orchestrated by the bifunctional Rel enzyme with synthetase and hydrolase activity and the two synthetases SasA/RelP and SasB/RelQ. Here we will summarize recent studies supporting the role of (p)ppGpp in the development of antibiotic resistance and tolerance as well as survival under adverse environmental conditions in Firmicutes. We will also discuss the impact of elevated (p)ppGpp levels on the development of persister cells and the establishment of persistent infections. (p)ppGpp levels are usually tightly controlled to allow optimal growth under non-stressed conditions. Upon the onset of certain ‘stringent conditions’ the sudden increase in (p)ppGpp levels limits growth while exerting protective effects. In Firmicutes, the (p)ppGpp-mediated restriction of GTP accumulation is one major mechanism of protection and survival under stresses such as antibiotic exposure.

## Teaser

An overview of the contribution of the stringent response and (p)ppGpp to the development of antibiotic resistance, tolerance, and persistence, as well as to survival under adverse environmental conditions in Firmicutes.

## Introduction

The stringent response or stringent control is a bacterial response to different forms of stress and is characterized by the synthesis of the messenger molecules pppGpp and ppGpp (hereafter referred to as (p)ppGpp). (For recent reviews, see Zhu et al. 2019, Das and Bhadra 2020, Fernandez-Coll and Cashel 2020, Pacios et al. 2020, Sinha et al. 2020, Steinchen et al. 2020, Anderson et al. 2021, Bange et al. 2021, Irving et al. 2021, Pulschen et al. 2021). One main feature of the stringent control is to keep macromolecular synthesis tightly coupled to nutrient availability and growth rate. Accordingly, (p)ppGpp can specifically block replication, translation, and transcription (Steinchen et al. 2020, Anderson et al. 2021, Irving et al. 2021). However, the downstream events that follow (p)ppGpp accumulation may vary among species and result in distinct phenotypic outcomes. There are major differences between the stringent response initially characterized in *Escherichia coli* and the response in Firmicutes. Differences can be seen not only in the phenotypic consequences of (p)ppGpp accumulation but also in the main players involved in the synthesis and degradation of the messengers. The role of (p)ppGpp in bacterial survival, antibiotic resistance, and tolerance as well as persister cell formation has mostly been analysed in Proteobacteria. Here we will discuss whether and to what extent this holds true for Firmicutes.

We will employ the recent consensus definitions to discriminate between antibiotic resistance, tolerance, and persistence (Balaban et al. 2019). Antibiotic resistance is characterized by a higher minimal inhibitory concentration (MIC) due to the genetic carriage of a resistance factor. Antibiotic-resistant bacteria not only survive otherwise lethal doses of antibiotics but can also replicate in the presence of antibiotics.

In contrast, antibiotic tolerance describes the ability to survive transient exposure to lethal concentrations of antibiotics without exhibiting an elevated MIC. Further, antibiotic-tolerant cells not only survive antibiotic treatment but can regrow after the removal of the antibiotic. Antibiotic tolerance can be detected by the overall slower killing of the whole population in time-kill assays and can be quantified by the metric minimum duration for killing (MDK) or by the TDtest, a modified disk-diffusion assay that enables the semi-quantitative evaluation of tolerance level (Gefen et al. 2017, Kotková et al. 2019).

The term antibiotic persistence describes a subpopulation of bacterial persister cells that survives intensive bactericidal antibiotic treatment without being antibiotic resistant, whereas the majority of the initial population is antibiotic susceptible and therefore rapidly killed. This phenomenon is characterized by a biphasic killing curve and therefore antibiotic tolerance and persistence can be distinguished in a time-kill assay (Brauner et al. 2016). Bacterial persister cells are phenotypic variants that enter a dormant or slow-growing state and become transiently antibiotic-tolerant (Lewis 2007, Balaban et al. 2019). Hereof the term ‘persistent infections’ needs to be distinguished, meaning difficult-to-treat infections that are not cleared by the host immune system. Persistent pathogens are difficult to eradicate with standard antimicrobial therapy as they deploy many regulatory strategies e.g. the stringent response, to compensate unfavourable host environmental conditions.

## (p)ppGpp making, breaking, and function in Firmicutes

### (p)ppGpp making

(p)pGpp is synthesized by RelA-SpoT-homologs (RSH), which catalyse the transfer of pyrophosphate originating from ATP to the 3´ OH group of GTP, GDP, or GMP. Long RSH enzymes, characterized by an N-terminal enzymatic domain bearing distinct motifs for (p)ppGpp synthetase or hydrolase activity and a C-terminal regulatory domain, are present in nearly all bacteria and display both (p)ppGpp hydrolytic and biosynthetic activity (Atkinson et al. 2011, Bange et al. 2021). Firmicutes possess one long RSH enzyme, Rel. Amino acid limitation through the accumulation of uncharged tRNAs is so far the only common condition that has been shown to induce the (p)ppGpp synthetase activity of Rel (Geiger et al. 2010, Pausch et al. 2020). Rel can be allosterically activated by (p)ppGpp (Roghanian et al. 2021), and its activity can be modulated by the second messenger c-di-AMP (Corrigan et al. 2015, Peterson et al. 2020, Krüger et al. 2021, Heidemann et al. 2022). In some Firmicutes, Rel directly interacts with the c-di-AMP-binding protein DarB, thereby linking potassium deprivation with a stringent response (Krüger et al. 2021, Ainelo et al. 2023). In *Bacillus subtilis*, lipid starvation (Pulschen et al. 2017), heat shock (Schafer et al. 2020), or certain antibiotics (Yang et al. 2022) can also induce Rel-dependent (p)ppGpp synthesis by so far ill-defined mechanisms.

In addition to the long RSH Rel, Firmicutes possess one or two small alarmone synthetases (SAS), SasA/RelP and SasB/RelQ. The (p)ppGpp synthesis by SAS enzymes is induced through transcriptional activation of the encoding genes and/or post-translational modifications. For example, RelP and RelQ from *Staphylococcus aureus* are part of the VraRS cell-wall stress regulon and are induced after vancomycin treatment (Geiger et al. 2014). In *B. subtilis*, phosphorylation of the response regulator WalR controls the heterogeneous expression of RelP (Libby et al. 2019), while in *Clostridioides difficile, relQ* transcription is induced by sublethal concentrations of different antibiotics (clindamycin, metronidazole, vancomycin, and fidaxomicin) (Pokhrel et al. 2020, Poudel et al. 2022). Post-translational activation/inhibition of SAS proteins is also important for function. RelQ from *B. subtilis* and *Enterococcus faecalis* form tetramers (Steinchen et al. 2015, Beljantseva et al. 2017). RelQ activity is strongly inhibited through the binding of single-stranded RNA (ssRNA). (p)ppGpp binding leads to disassociation of the RelQ: RNA complex and activation of RelQ (Beljantseva et al. 2017). RelP activity is inhibited by both pppGpp and ppGpp, activated by Zn^2+^, and insensitive to inhibition by RNA (Manav et al. 2018, Steinchen et al. 2018). Whereas in *S. aureus*, RelQ seems to be insensitive to ssRNA, and RelP is not allosterically stimulated by the addition of pppGpp, ppGpp, or pGpp (Yang et al. 2019). In this bacterium, RelQ is mainly held in an inactive state when overexpressed (Horvatek et al. 2020). Thus, the expression and activity of RelP and RelQ seem to differ considerably between species, although these two proteins are highly homologous. Post-translational regulatory mechanisms are likely important to fine-tune (pp)pGpp synthesis under different growth conditions.

### (p)ppGpp breaking

Rel is so far the only described enzyme to degrade (p)ppGpp in Firmicutes and thus important to reset the system. Under non-inducing conditions, Rel is primarily in a hydrolase-on/synthetase-off conformation (Gratani et al. 2018), while starved ribosomes are sufficient to inhibit the hydrolytic activity (Takada et al. 2021). The strong hydrolase activity of Rel from *S. aureus* makes the enzyme essential for the detoxification of (p)ppGpp produced by RelP or RelQ (Geiger et al. 2010). In *B. subtilis and E. faecalis, rel* mutants are viable but severely impaired in growth due to (p)ppGpp overproduction (Srivatsan et al. 2008, Gaca et al. 2013, Ababneh and Herman 2015). The hydrolase NahA of *B. subtilis* efficiently hydrolyses (p)ppGpp to produce pGpp, thereby modulating alarmone composition and function (Yang et al. 2020). The results indicate the existence and physiological relevance of pGpp as a third alarmone, with functions that can be distinct from those of (p)ppGpp.

### Targets of (p)ppGpp

In gram-positive pathogens, the stringent response plays important roles in virulence (for reviews, see Kundra et al. 2020) and antibiotic tolerance (discussed below). The global transcriptional effects of (p)ppGpp have been examined previously in several Firmicutes, such as *B. subtilis* (Eymann et al. 2002), *Streptococcus pneumoniae* (Kazmierczak et al. 2009), *E. faecalis* (Gaca et al. 2012), *Streptococcus mutans* (Nascimento et al. 2008), and *S. aureus* (Geiger et al. 2012, Horvatek et al. 2020). Many of the observed downstream effects are mediated via specific binding of (p)ppGpp to target proteins resulting in inhibition of replication (DnaG), ribosome function (RsgA, RbgA, Der, Era, HflX, and ObgE), secretion (Ffh and FtsY), and GTP synthesis (GuaB, HprT, Gmk, GdpP, Pde, PgpH, and PurR) (for reviews, see Steinchen et al. 2020, Anderson et al. 2021, Zegarra et al. 2023). Moreover, (p)ppGpp can control transcription via (p)ppGpp-specific riboswitches (Sherlock et al. 2018).

**Figure 1.**
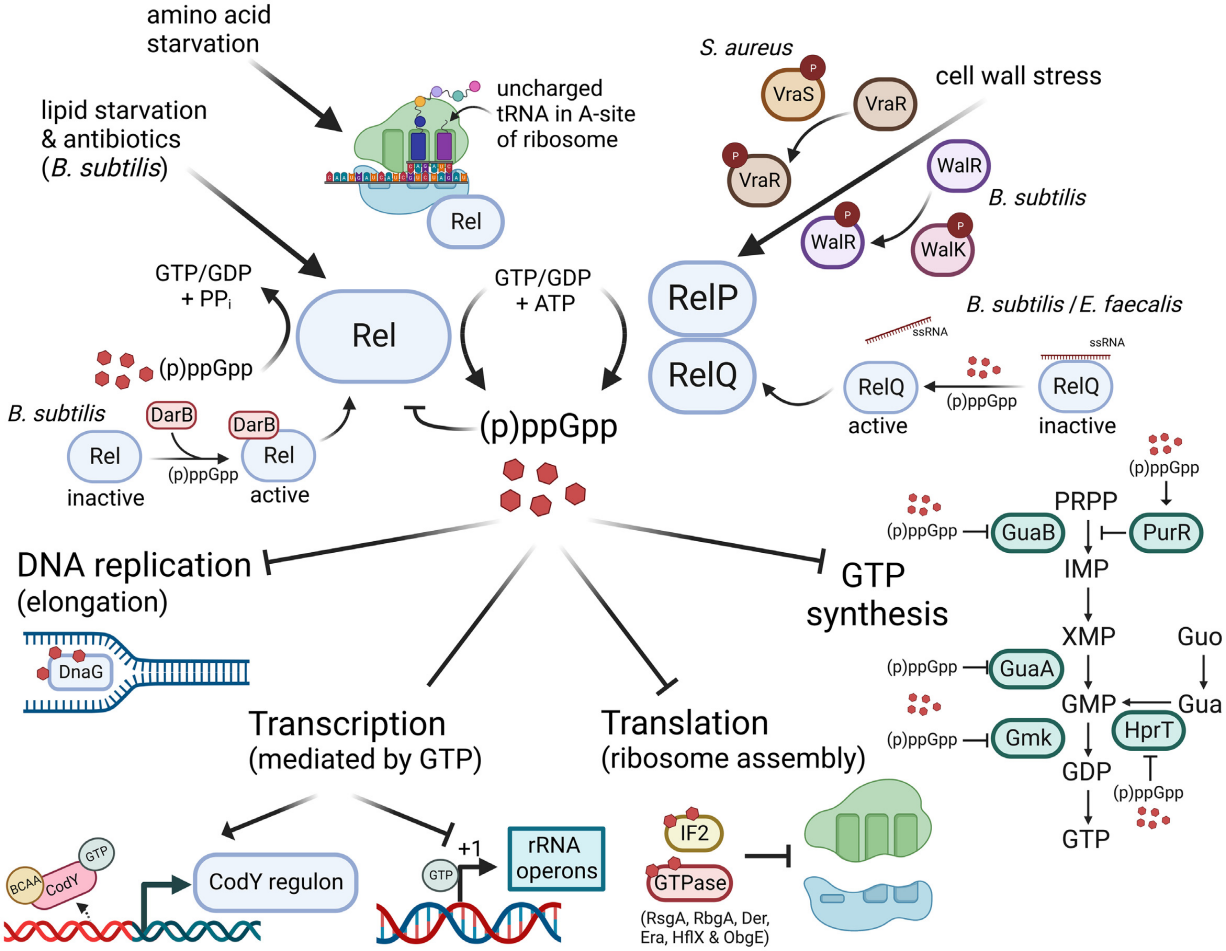
Activation of the stringent response, synthesis of (p)ppGpp, and main effects of (p)ppGpp on bacterial physiology in Firmicutes. Rel-dependent (p)ppGpp synthesis is induced during amino acid starvation (by sensing of stalled ribosomes by Rel), but in *B. subtilis* also during lipid starvation, heat shock, and antibiotic stress. RelP and RelQ are activated during cell wall stress by the two-component systems VraRS in *S. aureus* or WalKR in *B. subtilis*. RelQ is also activated via the (p)ppGpp-induced release of an inactivating ssRNA. The substrates for synthesis are GTP or GDP and ATP, and the reaction is catalyzed by the synthetase domain of Rel and SAS enzymes (RelP/RelQ). (p)ppGpp degradation occurs solely by the hydrolase domain of Rel. Accumulation of (p)ppGpp modulates then bacterial physiology by directly binding to a variety of enzymes or via lowering of the GTP pools. Low intracellular levels of GTP and BCAA relieve the repression of the transcriptional regulator CodY. In addition, GTP depletion represses the transcription of genes, e.g. rRNA operons, requiring GTP as their initiating nucleotide. Created with BioRender.com.

Of note, in contrast to *E. coli*, (p)ppGpp in Firmicutes does not interfere with RNA polymerase activity (Hauryliuk et al. 2015). Instead, in these bacteria, (p)ppGpp regulates transcription indirectly by strongly lowering the intracellular GTP pool (Krasny et al. 2008, Geiger et al. 2012, Kriel et al. 2012, Kastle et al. 2015). A decrease in the GTP levels leads to the repression of nucleotide-sensitive, GTP-initiating promoters, e.g. those of rRNA genes (Krasny and Gourse 2004, Kastle et al. 2015). Low GTP levels also affect the CodY regulon in most Firmicutes. The transcription factor CodY, when loaded with GTP and branched-chain amino acids, acts mainly as a repressor of many genes involved in amino acid synthesis and virulence (Pohl et al. 2009, Majerczyk et al. 2010, Geiger and Wolz 2014). However, GTP is not a co-factor for CodY in streptococci (Zhu et al. 2019). Recently, it was shown that (p)ppGpp also targets the transcriptional regulator PurR, which adds a further mechanism how the alarmone can modulate transcription (Anderson et al. 2021). The mechanism of activation of the stringent response and the main effect of (p)ppGpp on bacterial physiology in Firmicutes are summarized in Fig. 1.

## (p)ppGpp and antibiotic resistance

### (p)ppGpp and Β-lactam resistance in MRSA

Antibiotic resistance represents a growing threat to global health, and the clinical burden of infections caused by antimicrobial-resistant pathogens is immense. Hereby, methicillin-resistant *S. aureus* (MRSA) is of particular concern. In the last few years, several groups have observed connections between methicillin resistance in *S. aureus* and the stringent response (Table 1). Methicillin resistance is conferred by the presence of the *mecA*/*mecC* gene that encodes the penicillin-binding protein PBP2A, a peptidoglycan transpeptidase with extremely low affinity for methicillin and other β-lactam antibiotics (Hartman and Tomasz 1984). Mupirocin inhibits the bacterial isoleucyl tRNA synthetase and, as such, induces Rel-dependent (p)ppGpp synthesis. Mupirocin treatment of MRSA strain N315 in combination with the β-lactam antibiotic oxacillin increases the expression of *mecA, pbpB*, and *pbpD* and confers resistance towards oxacillin (Aedo and Tomasz 2016). Further, induction of the stringent response converted an initially heterogeneous population with only a small subset of bacterial cells extremely resistant to β-lactams into a homogeneous and highly resistant population (Kim et al. 2013, Aedo and Tomasz 2016). Mwangi et al. used mutant selection and whole genome sequencing to identify two point mutations in *rel* that conferred high-level resistance (Mwangi et al. 2013). Both mutations impaired the hydrolase activity of Rel and thus increased (p)ppGpp levels. The mutant phenotype could be partially rescued by additional loss-of-function mutations within *relQ*. These data indicate that (p)ppGpp produced by either Rel or RelQ can result in enhanced oxacillin resistance. In strain N315 *relQ* deletion alone did not affect the level of β-lactam resistance (Matsuo et al. 2019). However, overexpression of *relQ* did result in an elevated MIC for oxacillin. In *Streptococcus pneumoniae*, inhibition of (p)ppGpp synthesis as well as other interactions in purine metabolism lead to reduced β-lactam and glycopeptide sensitivity manifested by increased relative growth (Leshchiner et al. 2022). In summary, an impact of (p)ppGpp on oxacillin resistance is supported by several studies.

**Table 1.** (p)ppGpp mediated antibiotic resistance, tolerance, and persistence in Firmicutes.

	Organism	Genotype/Method of induction	Phenotype	Literature
Antibiotic resistance	*S. aureus*	Upregulation of *mecA* expression during mupirocin treatment (SR induction)	Resistance to β-lactam antibiotics	(Kim et al. 2013, Aedo and Tomasz 2016)
	*S. aureus*	Increased (p)ppGpp synthase activity of RelQ/decreased (p)ppGpp hydrolase activity of Rel due to point mutations	Increased (p)ppGpp levels are associated with oxacillin resistance	(Mwangi et al. 2013)
	*S. aureus*	*ΔrelQ*	Reduced resistance to β-lactam antibiotics	(Bhawini et al. 2019)
	*S. aureus*	*relQ* overexpression	Increased MIC for oxacillin	(Matsuo et al. 2019)
	*E. faecalis*	*ΔrelQ, ΔrelAQ*	Decreased MIC for vancomycin	(Abranches et al. 2009)
	*S. aureus*/*S. pneumoniae*	*Δrel_syn_*	Decreased MIC for mupirocin	(Kazmierczak et al. 2009, Geiger et al. 2010)
	*B. subtilis*	*ΔvmIR*/(p)ppGpp^0^	Decreased MIC for tiamulin, retapamulin, lincomycin, iboxamycin, and virginiamycin M1	(Takada et al. 2022)
	*S. aureus*	Blocking of (p)ppGpp with MIP-NP	Increased efficiency rates for kanamycin and tetracycline	(Chen et al. 2022)
	*B. subtilis*	*Δrel_syn_*, (p)ppGpp^0^	Bacteriostatic antibiotics chloramphenicol and tetracycline become bactericidal, decreased MIC for (p)ppGpp^0^	(Yang et al. 2022)
	*B. subtilis*	*Δrel*, (p)ppGpp^0^	Cerulenin treatment is bactericidal	(Pulschen et al. 2017)
	*S. aureus*	*Δrel_syn_*, (p)ppGpp^0^	Mupirocin treatment is bactericidal	(Geiger et al. 2010, Geiger et al. 2014)
Antibiotic tolerance	*S. aureus*	Mupirocin treatment	Tolerance to vancomycin	(Singh et al. 2017)
	*S. aureus*	*ileS* mutation	Tolerance to vancomycin, imipenem, and daptomycin	(Singh et al. 2017)
	*S. aureus*	*ΔrelPQ* and (p)ppGpp^0^	Decreased tolerance to vancomycin and ampicillin	(Geiger et al. 2014)
	*E. faecalis*	*ΔrelQ, Δrel ΔrelQ*	Decreased tolerance to vancomycin	(Abranches et al. 2009)
	*E. faecalis/B. subtilis*	*Δrel*	Enhanced tolerance to vancomycin	(Abranches et al. 2009, Yang et al. 2022)
		(p)ppGpp^0^	Decreased tolerance to vancomycin after chloramphenicol pretreatment	
	*E. faecalis*	*ΔrelQ* → low (p)ppGpp	Decreased survival in presence of norfloxacin and ampicillin	(Gaca et al. 2013)
	*S. aureus*	Nonsense mutation in *rel* → high (p)ppGpp	Enhanced tolerance to ciprofloxacin	(Matsuo et al. 2019)
	*S. aureus*	Point mutation in *rel*	Enhanced tolerance to daptomycin	(Berti et al. 2018)
	*E. faecium*	Point mutation in *rel*	Tolerance to vancomycin, linezolid, and daptomycin in biofilm	(Honsa et al. 2017)
	*S. aureus*	*ΔrsgA* or *rsgA* mutation	Tolerance to β-lactam antibiotics, vancomycin and ciprofloxacin	(Corrigan et al. 2016)
	*C. difficile*	Inhibition of Rel activity by (p)ppGpp analaog relacin or antisense *rel* RNA	Reduced tolerance to clindamycin and metronidazole	(Pokhrel et al. 2020)
	*B. subtilis/E. faecalis*	(p)ppGpp^0^, eleveated intracellular GTP levels	Bactericidal effect of chloramphenicol and tetracycline	(Yang et al. 2022)
	*S. aureus*	Triclosan treatment	Increased tolerance to ampicillin, kanamycin, streptomycin, and ciprofloxacin	(Westfall et al. 2019)
	*S. aureus*	(p)ppGpp^0^	Decreased tolerance to vancomycin in biofilm, increased resolution of biofilm in presence of vancomycin	(Salzer et al. 2020)
	*S. aureus*	(p)ppGpp^0^	Increased biofilm and planktonic culture antibiotic tolerance to ciprofloxacin and vancomycin	(Walsh et al. 2023)
	*E. faecium*	relA mutation → elevated (p)ppGpp	Increased biofilm antibiotic tolerance to linezolid and daptomycin	(Honsa et al. 2017)
Persistence	*S. aureus*	*Δrel_syn_*, (p)ppGpp^0^	(p)ppGpp is not required for persister formation	(Conlon et al. 2016)
	*B. subtilis*	Multisite phosphorylation leads to phenotypic variation in *sasA* expression levels	Increased tolerance to ciprofloxacin in cells expressing high *sasA* levels	(Libby et al. 2019, Diez et al. 2020, Diez et al. 2022)
	*B. subtilis*	GTP depletion by (p)ppGpp induction	Persistence to vancomycin and ciprofloxacin (tolerance in subpopulation of cells with GTP depletion)	(Fung et al. 2020)

### (p)ppGpp-defective bacteria show decreased MIC for diverse antibiotics

(p)ppGpp also influences the sensitivity of bacteria to other antibiotics (Table 1). A (p)ppGpp^0^ (common nomenclature for a strain in which all synthetases are mutated) strain of *E. faecalis* showed enhanced sensitivity not only towards the cell-wall targeting antibiotic vancomycin (Abranches et al. 2009), but also to the bactericidal drugs norfloxacin, ampicillin, and chloramphenicol (Gaca et al. 2013). *S. aureus* and *S. pneumoniae rel* mutants were shown to display a significantly higher susceptibility towards mupirocin than the parental strains (Kazmierczak et al. 2009, Geiger et al. 2010). It is assumed that upregulation of genes involved in isoleucine biosynthesis in the wildtype but not in the mutants increases the intracellular levels of isoleucine, which antagonizes mupirocin and thereby enhances the MIC (Geiger et al. 2010).

In *B. subtilis*, (p)ppGpp-mediated signalling was shown to enhance the expression of the ABCF ATPase VmIR—a resistance factor conferring resistance to the translation inhibitors pleuromutilin, linocsamide, and type A streptogramin—during challenge with VmIR-cognate antibiotics (Takada et al. 2022). Significant inhibition of translation is the key to VmlR induction. Accordingly, VmlR mainly functions during the stationary phase, when nutrients are scarce, and bacteria accumulate (p)ppGpp. (p)ppGpp accumulation reduces translation initiation by binding of (p)ppGpp to the initiation factor 2 (IF2) and associated GTPases (Corrigan et al. 2016, Diez et al. 2020). Thus, genetic disruption of *vmIR* or the (p)ppGpp synthesis pathway caused sensitivity to the pleuromutilin tiamulin, lincosamides such as the natural product lincomycin, as well as the more potent semi-synthetic antibiotic clindamycin, the recently developed fully synthetic iboxamycin, and the type A streptogramin virginiamycin M1 (Takada et al. 2022). Moreover, specific binding of molecularly imprinted nanoparticles (MIP-NP) to (p)ppGpp led to a significant increase in antibiotic efficiency rates (kanamycin, oxytetracycline) both in *E. coli* and *S. aureus* (Chen et al. 2022).

### (p)ppGpp is needed to prevent the bactericidal effect of antibiotics

Not only is the MIC of a variety of antibiotics lower for (p)ppGpp-defective bacteria, but there have also been several studies showing that antibiotics that exhibit bacteriostatic activity in wild-type cells become bactericidal in (p)ppGpp-deficient cells (Table 1) (Kazmierczak et al. 2009, Geiger et al. 2010, Gaca et al. 2013, Pulschen et al. 2017, Yang et al. 2022). For example, Yang et al. not only observed a decreased MIC for the bacteriostatic antibiotics chloramphenicol and tetracycline but also the bactericidal activity of these antibiotics in (p)ppGpp-defective *B. subtilis* (Yang et al. 2022), similar to what was observed for cerulenin treatment (Pulschen et al. 2017).

Altogether, increased antibiotic resistance towards several antibiotics was observed to correlate with high (p)ppGpp concentrations. (p)ppGpp-mediated resistance might be conferred by upregulation of resistance genes, e.g. *mecA* (Aedo and Tomasz 2016), slow growth, e.g. via inhibition of translation (Corrigan et al. 2016), and/or (p)ppGpp related changes in the nucleotide pool. The latter is supported by the observation that purine availability and metabolism, which are unbalanced in (p)ppGpp-defective *S. aureus*, can control antibiotic susceptibility towards oxacillin (Nolan et al. 2022). Further studies are needed to clarify which of these putative mechanisms is responsible for the antibiotic resistance observed in the different cases.

## (p)ppGpp and antibiotic tolerance

Antibiotic tolerance is an important contributor to antibiotic treatment failure, but it also acts as a precursor to antibiotic resistance (Levin-Reisman et al. 2019, Windels et al. 2019). As most antibiotics target active metabolic processes, a decreased growth rate and reduced metabolic activity contribute to the development of multidrug tolerance. (p)ppGpp leads to an immediate shut-down of replication and translation (for reviews, see Sinha et al. 2020, Zegarra et al. 2023), and high (p)ppGpp levels likely contribute to antibiotic tolerance. This assumption is supported by the following studies (Table 1).

### Contribution of small alarmone synthetases (SAS) to antibiotic tolerance

In *S. aureus, relP*, and *relQ* expression is specifically induced after treatment with the cell-wall active antibiotics ampicillin and vancomycin (Geiger et al. 2014). The elevated (p)ppGpp levels in turn protect from antibiotic killing, since a *relPQ* double mutant showed a reduced survival rate upon treatment with these antibiotics, but no difference in MIC between wild-type and *relPQ* mutants could be observed (Geiger et al. 2014). Likewise, mutation of *ileS*, encoding the isoleucyl-tRNA synthetase, leads to Rel-dependent (p)ppGpp synthesis and again increased vancomycin tolerance (Singh et al. 2017). Similar observations were reported for *E. faecalis*. In growth curve experiments with sub-inhibitory concentrations of vancomycin, enhanced tolerance was observed in a *relA* mutant strain that exhibited higher (p)ppGpp levels due to missing hydrolase activity (Abranches et al. 2009). By contrast, a *relQ* mutant strain grew slower than its parental strain and was more rapidly killed in time-kill experiments using a 5x MIC of vancomyin. In *B. subtilis*, chloramphenicol treatment resulted in Rel-dependent (p)ppGpp synthesis and thereby increased tolerance to vancomycin (Yang et al. 2022).

The impact of (p)ppGpp on tolerance towards other antibiotics is less clear. In *E. faecalis*, a (p)ppGpp^0^ (*rel, relQ*) strain is more sensitive to killing by norfloxacin and ampicillin (Gaca et al. 2013). In methicillin-sensitive *S. aureus* (MSSA), mutations of *rel* that likely lead to higher (p)ppGpp accumulation and an overall stringent-response-like transcription pattern have been linked to ciprofloxacin tolerance (Matsuo et al. 2019). However, Geiger et al. did not observe ciprofloxacin tolerance in *S. aureus* mutants with defective (p)ppGpp synthetases (Geiger et al. 2014). During prolonged daptomycin exposure, *S. aureus* acquired several mutations, one of which was within *rel* (L61F). A *de novo*-generated strain carrying the sole mutation L61F in Rel was less susceptible to daptomycin in time-kill assays (Berti et al. 2018). Of note, it is not clear whether the mutation made the bacteria produce more or less (p)ppGpp. Chemical inhibition of *rel* by the (p)ppGpp analog relacin or transcriptional repression by antisense *rel* RNA reduced tolerance to clindamycin and metronidazole (Pokhrel et al. 2020). Very recently, Yang et al. showed that *B. subtilis* and *E. faecalis* produce (p)ppGpp in response to chloramphenicol treatment through *rel*, which protected the bacteria from antibiotic killing (Yang et al. 2022). The protective effect could be linked to the decreased intracellular GTP pool. Elevated GTP levels in a (p)ppGpp^0^ strain promoted the bactericidal effect of chloramphenicol. The widely used antimicrobial triclosan is a bacteriostatic inhibitor of fatty acid synthesis and has been shown to increase tolerance to bactericidal antibiotics in *E. coli* and MRSA (Westfall et al. 2019). Very recently, it has been shown that triclosan treatment induces antibiotic tolerance to ciprofloxacin and vancomycin via the stringent response in *S. aureus* planktonic cultures and biofilms (Walsh et al. 2023).

### Biofilm-related antibiotic tolerance

Biofilms are normally more tolerant to high concentrations of antibiotics than planktonic cultures. In *S. aureus*, we could show that vancomycin-induced *relPQ* expression contributes to biofilm formation and biofilm-related antibiotic tolerance. Thus, RelP and RelQ play a major role in biofilm formation and maintenance under cell wall stress conditions (Salzer et al. 2020). A persistent vancomycin-resistant *Enterococcus faecium* (VRE) isolate harboured a *rel* mutation, resulting in elevated (p)ppGpp levels (Honsa et al. 2017). The strain showed tolerance towards linezolid and daptomycin when growing in biofilm but remained susceptible in MIC testing and during planktonic growth.

In summary, several studies indicate that elevated (p)ppGpp, either through mutations of the Rel hydrolase or via activation of synthetases, can contribute to antibiotic tolerance without necessarily conferring resistance. However, (p)ppGpp is likely not the only or even the main trigger for bacterial tolerance.

## (p)ppGpp and formation of persister cells

The molecular mechanisms underlying persister cell formation are highly debated, and the role of (p)ppGpp in this process remains unclear (Balaban et al. 2013, Kaldalu et al. 2016, Kaldalu et al. 2020, Pacios et al. 2020, Song and Wood 2020, Fernandez-Garcia et al. 2022, Verstraete et al. 2022). Persister cells are thought to be ‘dormant’ subpopulations that can either be carried over from the stationary phase (Type I) or formed by stochastic switching during exponential growth (Type II) (Balaban 2011). The current standard method for persister identification at the population level is a biphasic killing curve. In many studies claiming to analyse persisters such a two-phase kill curve is not demonstrated, hampering the interpretation of many findings. The term ‘triggered persistence’ indicates a form of persistence that is induced by particular signals. Persistence seems to be an evolvable trait, indicating the existence of active mechanisms that form and maintain the persister phenotype (Wilmaerts et al. 2019, Verstraete et al. 2022). However, it was also assumed that persistence arises as a result of stress and errors (PASH hypothesis-Persistence as stuff happens) rather than ‘stochastically’ as a form of ‘bet hedging’ (Levin et al. 2014, Fernandez-Garcia et al. 2022).

### (p)ppGpp heterogeneity on the single cell level

Nevertheless, genetic factors can impact persistence levels. (p)ppGpp and toxin/antitoxin systems were discussed as major triggers for dormancy/persistence, at least in Proteobacteria (Korch et al. 2003, Amato et al. 2013, Helaine et al. 2014, Chowdhury et al. 2016, Svenningsen et al. 2019, Pontes and Groisman 2020, Urbaniec et al. 2022, Verstraete et al. 2022). However, the low frequency combined with the transient nature of persister cells makes them very challenging to study. Some models assume that stochastic (p)ppGpp synthesis in a very small number of cells leads to persistence. So far, it has not been possible to measure the levels of (p)ppGpp in single cells, and such assumptions are based on the stochastic expression of stringently regulated marker genes. However, most of the marker genes are also highly regulated independently of (p)ppGpp. The first paper describing such stochastic (p)ppGpp-dependent regulation in *E. coli* has been retracted (Maisonneuve et al. 2018), and the assumption has been challenged by several groups (Ramisetty et al. 2016, Liu et al. 2017, Goormaghtigh et al. 2018). Internalization of *Salmonella* by macrophages induces phenotypic heterogeneity, leading to the formation of non-replicating persisters, which could serve as a reservoir for relapsing infection. Deletion of *relA* and *spoT* resulted in decreased persister numbers, supporting that (p)ppGpp contributes to persistence under these specific conditions. However, in many species, (p)ppGpp mutants are often severely impaired in many processes and often gain compensatory mutations. Thus, these studies were questioned for their ability to conclusively determine the involvement of (p)ppGpp in persister cell formation (Kaldalu et al. 2020, Pontes and Groisman 2020).

It was also proposed that inactivation of ribosomes via (p)ppGpp-dependent dimerization is a general mechanism leading to persistence (Song and Wood 2020, Fernandez-Garcia et al. 2022). In *E. coli*, (p)ppGpp stimulates the expression of hibernation factors to dimerize 70S ribosomes into 100S ribosomes (Song and Wood 2020). Ribosome dimerization protects ribosomal proteins and rRNA from degradation while allowing rapid recovery from stress upon dimer dissociation. For *E. coli*, it was shown that such resuscitation from the dormant state is heterogeneous, and persistence decreases in the absence of (p)ppGpp. However, (p)ppGpp did not affect the resuscitation of single-cell persisters (Chowdhury et al. 2016, Kim et al. 2018).

### Is there evidence for a role of (p)ppGpp in persister formation in Firmicutes?

In *S. aureus*, persister formation following ciprofloxacin or gentamycin exposure does not require (p)ppGpp since a (p)ppGpp^0^ mutant shows a similar rate of persister formation (Conlon et al. 2016). Hibernation is controlled by one long hibernation-promoting factor (HFP) homologue and two translation factors. For resuscitation, 100S ribosomes are split synergistically by the action of ribosome-recycling factor (RRF), GTPase elongation factor G (EF-G), and HlfX (Gohara and Yap 2018). Expression of *hfp* and the recycling factor *hflX* is independent of (p)ppGpp (Horvatek et al. 2020). However, HflX was shown to bind (p)ppGpp (Corrigan et al. 2016). Furthermore, disassembly of the 100S ribosome strictly requires GTP hydrolysis (Basu and Yap 2017, Basu et al. 2020). Thus, the changing nucleotide pools upon stringent response are likely to impact 100S disassembly on the protein level and thus may contribute to rapid growth resumption. In *B. subtilis*, induction of a (p)ppGpp synthetase-encoding gene in a pppGpp^0^ strain resulted in 100 S ribosome formation (Tagami et al. 2012), further indicating that also in Firmicutes, (p)ppGpp is involved in hibernation and/or disassembly of 100 S ribosomes. However, whether such processes are stochastic and thus involved in persistent formation remains unclear.

In *B. subtilis*, there is evidence for stochastic (p)ppGpp synthesis. Cultures experiencing nutrient limitation contain distinct subpopulations of cells exhibiting either comparatively high or low protein synthesis activity. This heterogeneity is determined by (p)pGpp levels (Libby et al. 2019, Diez et al. 2020, Diez et al. 2022). *sasA* encoding the small synthetase SasA/RelP exhibits high levels of extrinsic noise in expression. Rare cells with unusually high levels of *sasA* expression display increased antibiotic tolerance. *sasA* expression depends on the transcription factor WalR, which is in turn regulated via multisite phosphorylation by the Ser/Thr kinase-phosphatase pair PrkC/PrpC and the histidine kinase WalK of the WalKR two-component system (Libby et al. 2019). Furthermore, the absence of any of the (p)ppGpp synthases results in the loss of bimodality. RelA and SasB/RelQ activities are necessary to generate the subpopulation of cells exhibiting low protein synthesis. Interestingly, SasA/RelP generates the subpopulation with high protein synthesis (Diez et al. 2022). This complex interaction is likely due to allosteric regulation of SasB/RelQ by the SasA/RelP product pGpp and the RelA product pppGpp.

It was also shown that in B*. subtilis*, a drop in GTP pools due to induction of the stringent response induces persister formation, and GTP was implicated as the major metabolic effector of (p)ppGpp-mediated persistence (Fung et al. 2020). Altogether, this complex interaction indicates that (p)ppGpp expression is indeed heterogeneous in *B. subtilis* and might thus contribute to the persister phenotype. Whether similar regulation occurs in other Firmicutes is less clear since, albeit highly homologous, the SAS enzymes can show major differences with regard to gene expression and allosteric regulation (Yang et al. 2019). For example, in *S. aureus, relP* and *relQ* are under the control of the VraRS two-component system (Geiger et al. 2014), whereas in *B. subtilis*, at least *relP* is controlled by the WalKR system (Libby et al. 2019). In *S. aureus* and *E. faecalis*, (p)ppGpp contributes to antibiotic tolerance (Abranches et al. 2009, Gaca et al. 2013, Geiger et al. 2014). Whether this indicates a role for (p)ppGpp in triggered persistence is less clear since clear biphasic kill curves were not provided in the above-mentioned studies.

## (p)ppGpp and survival in persistent infections

Whereas the role of (p)ppGpp in persister cell formation is still controversial, there is evidence that (p)ppGpp contributes to establish long-term (persistent) infections in diverse host niches. Of note, the term ‘persistent infection’ has to be clearly distinguished from ‘antibiotic persistence’ described above. Persistent infection is generally used to describe infections in the host that are not cleared by the host immune system, whereas antibiotic persistence describes a bacterial population that is refractory to antibiotic treatments (Brauner et al. 2016, Balaban et al. 2019). There are several studies indicating that elevated basal (p)ppGpp levels and/or induction of the stringent response *in vivo* contribute to the development of persistent infections.

### Increased (p)ppGpp synthesis promotes persistent infections

In *E. faecalis*, it has been observed that during prolonged bloodstream infection in an immunocompromized host, *relA* missense mutations arise that constitutively activate the stringent response. These mutations were associated with a fitness tradeoff but also increased antibiotic tolerance *in vivo* and delayed eradication in an immunocompromized host (Honsa et al. 2017). No change in MIC for several antibiotics, including those that had been administered to the infected patient was observed.

Likewise, after *S. aureus* persistent infection a strain with higher (p)ppGpp levels due to mutations of *rel* (F128Y) was isolated (Gao et al. 2010). Elevated (p)ppGpp levels increased resistance to β-lactam antibiotics and provided a significant fitness advantage in presence of bactericidal antibiotics as a result of (p)ppGpp-mediated reduced growth (longer lag time and/or lower growth rate) (Bryson et al. 2020, Deventer et al. 2022). Moreover, even strains from persistent bacteremia without mutation within *rel, relP*, or *relQ* were shown to display significantly higher (p)ppGpp levels compared to MRSA isolates from resolving bacteremia (Li et al. 2020). The persistent strains also showed high expression of the virulence peptides phenol-soluble modulins (PSMs), higher polymorphonucleate (PMN) lysis and survival, and endothelial cell adherence, which were linked to the elevated (p)ppGpp levels. The molecular basis for the upregulation of (p)ppGpp in these strains remains unknown.

Collectively, in *S. aureus* as well as in *E. faecalis* an increase in (p)ppGpp levels has been linked to more chronic persistent infections. The mutations might have been selected due to their immune evasion properties or their higher antibiotic tolerance phenotype.

## (p)ppGpp growth control and survival *in vitro*

(p)ppGpp is a major regulator of growth-rate control in *E. coli* (Potrykus et al. 2011, Ahn-Horst et al. 2022). However, the effects of (p)ppGpp on global protein synthesis and survival to starvation seem to be highly dependent on the conditions (N- vs C-limitation) (Iyer et al. 2018). In *S. aureus*, pppGpp^0^ mutants are still able to inhibit rRNA synthesis in the later growth phase (Kastle et al. 2015). This indicates that, at least in *S. aureus*, (p)ppGpp-independent mechanisms also account for growth-rate control when nutrients become limited. (p)ppGpp seems to have different roles depending on its abundance in the cell (Fernandez-Coll and Cashel 2020). The role of (p)ppGpp in bacterial survival under non-stressed conditions, i.e. when the levels of (p)ppGpp are low, is less clear. It was proposed that basal levels of (p)ppGpp may have different effects than high level bursts during stressful situations (Fernandez-Coll and Cashel 2020). Several binding partners of (p)ppGpp have been uncovered, displaying dissociation constants from below one micromolar to more than one millimolar. Nucleotide metabolism enzymes are among the first targets to be affected by rising (p)ppGpp, while more fundamental processes such as DNA replication are among the last (Steinchen et al. 2020). (p)ppGpp^0^ strains in Firmicutes usually show no growth defect under non-stressed conditions, e.g. in rich medium (Abranches et al. 2009, Geiger et al. 2010, Kriel et al. 2012). However, (p)ppGpp^0^ mutants have altered transcriptomes, even under non-stressed conditions (Gaca et al. 2013), and basal levels of (p)ppGpp seem to be important to maintain GTP homeostasis in Firmicutes but not in Proteobacteria (Anderson et al. 2019). After shift to amino acid limiting conditions a sudden raise of (p)ppGpp is necessary to protect the bacteria from death (Geiger et al. 2010, Kriel et al. 2012, Bittner et al. 2014, Kriel et al. 2014). In *B. subtilis*, it was shown that controlling GTP accumulation is required for survival during amino acid starvation (Bittner et al. 2014). Manipulating the GTP levels in pppGpp^0^ mutants provided evidence that GTP regulates growth rate in a dose-dependent manner in *B. subtilis*. In the wildtype, (p)ppGpp restricts the upper limit of GTP to ensure optimal growth and survival.

Inhibition of fatty acid synthesis by cerulenin in *B. subtilis* was also found to be lethal only in the absence of (p)ppGpp synthesis (Pulschen et al. 2021). Adaptation to fatty acid starvation in wild-type bacteria requires the activity of the Rel synthetase, although (p)ppGpp levels remain below detection limit. Thus, (p)ppGpp-mediated balancing of the GTP/ATP ratio is essential for bacterial survival at least under certain conditions (fatty acid or amino acid starvation). Under such conditions, the GTP/ATP ratio in pppGpp^0^ strains remains high and probably keeps the cell growing, which will eventually perturb membrane function and lead to cell death.

In certain media, the survival of the *S. aureus* (p)ppGpp^0^ mutant is diminished in the stationary phase, which has also been linked to uncontrolled GTP accumulation (our unpublished data). In the stationary phase, *S. aureus* acquires tolerance towards oxidative stress by maintenance of redox and iron homeostasis, which is mediated by (p)ppGpp (Fritsch et al. 2020, Horvatek et al. 2020). Consistently, in the (p)ppGpp^0^ mutant, the antioxidant stress response genes were induced due to elevated ROS levels in the stationary phase (Fritsch et al. 2020). (p)ppGpp synthesis by the inducible synthetases resulted in the activation of ROS-inducing toxins and the simultaneous expression of detoxifying systems to protect the producer. Thereby, (p)ppGpp prepares the cells for survival to elevated ROS (Horvatek et al. 2020). Thus, in *S. aureus*, (p)ppGpp not only confers tolerance to antibiotics but also to ROS (Fritsch et al. 2020, Horvatek et al. 2020), although oxidative stress *per se* does not seem to induce (p)ppGpp synthesis.

## Conclusion and outlook

A tight control of (p)ppGpp level is crucial for bacterial growth and survival. This balance is achieved by the activity of three (p)ppGpp synthetases (Rel, RelP, and RelQ) and the strong hydrolase activity of Rel. Rel and RelQ-dependent (p)ppGpp synthesis is usually restricted and activated under certain conditions, including amino acid limitation and allosteric activation via (p)ppGpp. Under ‘stringent conditions’, (p)ppGpp contributes to active adaptation to stressful conditions mainly via up-regulation of certain genes, e.g. amino acid synthesis genes, oxidative stress response genes, and virulence genes. (p)ppGpp acts mainly as an inhibitory molecule (Anderson et al. 2021). High (p)ppGpp levels result in severe growth arrest, which is nevertheless beneficial for the bacteria to survive under certain conditions, e.g. antibiotic stress *in vitro* and during persistent infections *in vivo*. However, (p)ppGpp overproduction comes with a fitness disadvantage. Thus, (p)ppGpp levels are usually controlled to allow optimal growth under non-stressed conditions (basal level) but also to protect from sudden insults (stringent conditions). In Firmicutes, the (p)ppGpp-mediated restriction of GTP accumulation is at least one, if not the only, mechanism for protection. How uncontrolled GTP levels in pppGpp^0^ strains lead to cell death remains to be elucidated.

In recent years, there has also been growing evidence that the nature of the alarmone (pppGpp, ppGpp, or pGpp) influences the stringent phenotype. The affinity of these molecules towards different targets can differ considerably (Steinchen et al. 2020, Anderson et al. 2021). While ppGpp and pppGpp are well-established alarmones, it has long been reported that bacteria can also produce other nucleotide derivatives, such as pGpp and (p)ppApp (Anderson et al. 2021). The function of pGpp remains elusive since its occurrence and potential activity were only recently appreciated in Firmicutes (Gaca et al. 2015, Horvatek et al. 2020, Yang et al. 2020). pGpp can be directly produced by certain (p)ppGpp synthetases using GMP and ATP as substrates or, alternatively, by enzymatic conversion from (p)ppGpp by the Nudix hydrolase NahA.

In summary, (p)ppGpp is an important molecule that controls bacterial survival and virulence. Of note, there might be major differences also between the different Firmicute species with respect to (p)ppGpp activation pathways as well as down-stream effects, e.g. GTP dysregulation. However, comparative studies have so far been rare, and species-specific properties remain to be further elucidated. It seems likely that the machineries to synthesize and sense (p)ppGpp have evolved in the different species to optimize adaptation to the unique environmental niches.

Common to all species is that high (p)ppGpp levels are growth limiting and thereby contributing to antibiotic tolerance. To what extent (p)ppGpp synthesis facilitates persister formation is still questionable. It was argued that the (p)ppGpp signalling pathway could be a prime target to simultaneously combat bacterial virulence and resistance. Although clinical validation of (p)ppGpp as a new target for antimicrobials is still missing, several approaches towards this end have been devised that show promise (for reviews, see Pulschen et al. 2021).
